# Acute *Schistosoma mansoni* Infection Increases Susceptibility to Systemic SHIV Clade C Infection in Rhesus Macaques after Mucosal Virus Exposure

**DOI:** 10.1371/journal.pntd.0000265

**Published:** 2008-07-23

**Authors:** Agnès-Laurence Chenine, Ela Shai-Kobiler, Lisa N. Steele, Helena Ong, Peter Augostini, Ruijiang Song, Sandra J. Lee, Patrick Autissier, Ruth M. Ruprecht, W. Evan Secor

**Affiliations:** 1 Department of Cancer Immunology and AIDS, Dana-Farber Cancer Institute, Boston, Massachusetts, United States of America; 2 Harvard Medical School, Boston, Massachusetts, United States of America; 3 Division of Parasitic Diseases, National Center for Infectious Diseases, Centers for Disease Control and Prevention, Atlanta, Georgia, United States of America; 4 Department of Biostatistics and Computational Biology, Dana-Farber Cancer Institute, Boston, Massachusetts, United States of America; 5 Division of Viral Pathogenesis, Beth Israel Deaconess Medical Center, Boston, Massachusetts, United States of America; George Washington University, United States of America

## Abstract

**Background:**

Individuals living in sub-Saharan Africa represent 10% of the world's population but almost 2/3 of all HIV-1/AIDS cases. The disproportionate HIV-1 infection rates in this region may be linked to helminthic parasite infections that affect many individuals in the developing world. However, the hypothesis that parasite infection increases an individual's susceptibility to HIV-1 has never been prospectively tested in a relevant in vivo model.

**Methodology/Principal Findings:**

We measured whether pre-existing infection of rhesus monkeys with a parasitic worm would facilitate systemic infection after mucosal AIDS virus exposure. Two groups of animals, one consisting of normal monkeys and the other harboring *Schistosoma mansoni*, were challenged intrarectally with decreasing doses of R5-tropic clade C simian-human immunodeficiency virus (SHIV-C). Systemic infection occurred in parasitized monkeys at viral doses that remained sub-infectious in normal hosts. In fact, the 50% animal infectious (AID_50_) SHIV-C dose was 17-fold lower in parasitized animals compared to controls (*P*<0.001). Coinfected animals also had significantly higher peak viral RNA loads than controls (*P*<0.001), as well as increased viral replication in CD4^+^ central memory cells (*P* = 0.03).

**Conclusions/Significance:**

Our data provide the first direct evidence that acute schistosomiasis significantly increases the risk of *de novo* AIDS virus acquisition, and the magnitude of the effect suggests that control of helminth infections may be a useful public health intervention to help decrease the spread of HIV-1.

## Author Summary

To test the hypothesis that infection with helmiths may increase host susceptibility to infection with HIV-1, we quantified the amount of a clade C simian-human immunodeficiency virus needed to infect rhesus macaques that had acute *Schistosoma mansoni* infections. Compared to control animals exposed to virus alone, monkeys with schistosomiasis required exposure to 17-fold lower levels of virus to become infected. The schistosome-infected monkeys also had significantly higher levels of initial virus replication and loss of a certain subset of memory T cells, both predictors of a more rapid progression to immune dysfunction. These results suggest that worm infections may increase the risk of becoming infected with HIV-1 among individuals with viral exposures. Furthermore, they support the idea that control programs for schistosomiasis and perhaps other parasitic worm infections may also be useful in helping to reduce the spread of HIV/AIDS in developing countries where helminths are endemic.

## Introduction

Sub-Saharan Africa represents only 10% of the world's population but more than 62% of the world's HIV/AIDS cases [1]. While it remains controversial whether HIV transmission and/or disease progression in sub-Saharan Africa differ from what is observed in industrialized countries [2], one factor that may contribute to any exacerbation of HIV/AIDS is the high prevalence of parasitic worm infections, such as schistosomiasis [3],[4]. However, because it is not possible to directly test this hypothesis in humans, studies to date have evaluated in vitro exposure of cells to virus [5],[6], effects of schistosome infection on established viral infection [7],[8], or epidemiologic evaluations of the effect of praziquantel treatment on viral load [9]–[12]. None of these approaches address the issue of whether a helminth infection increases the susceptibility of the host to acquire *de novo* infection with an immunodeficiency virus after mucosal exposure, the predominant route of HIV transmission in humans.

In order to directly investigate this essential question, we tested the effect of *Schistosoma mansoni* infection on host susceptibility to immunodeficiency virus infection using schistosome-infected and control macaques exposed intrarectally to successively lower doses of the recently described R5-tropic SHIV-C, SHIV-1157ipd3N4 [13]. This strain is highly relevant for our study because an estimated 90% of all new HIV-1 infections occur by mucosal transmission, which almost exclusively involves R5 strains [14], and clade C strains cause >50% of all HIV-1 infections worldwide [1]. We also assessed the impact of schistosome infection on viral loads and immune cell profiles in monkeys with SHIV infection. These studies represent the first prospective evaluation of the impact of helminth infection on immunodeficiency virus transmission in a relevant model.

## Methods

### Animals

Chinese-origin adult female rhesus macaques were housed at the animal facility of the Centers for Disease Control and Prevention (CDC) in Atlanta, GA. Protocols were approved and animals were maintained in accordance with the guidelines of the Institution Animal Care and Use Committees for both CDC and the Dana-Farber Cancer Institute (DFCI). All procedures employed were consistent with the Guide for Care and Use of Laboratory Animals. Animals were free of helminth infection prior to our study.

### Virus

SHIV-1157ipd3N4 [13], an R5-tropic SHIV-C infectious molecular clone, is a monkey-adapted, late form of SHIV-1157i, which encodes most of the *env* sequences of a primary HIV-1 clade C strain isolated from a recently infected Zambian infant. SHIV-1157ipd3N4 was engineered with an additional NF-κB site per long terminal repeat (LTR) in order to enhance viral replication. The methodology used to construct this virus as well as its parental biologic strains are described by Song et al. [13]. Both the early and the late forms of our SHIV-C were pathogenic in rhesus monkeys, although disease progression was somewhat slow, with AIDS developing approximately 2.5–5.5 years post-inoculation.

### Rectal SHIV-1157ipd3N4 inoculation

Animals were anesthetized with ketamine and placed in a prone position. SHIV-1157ipd3N4 dilutions were prepared in RPMI in a total volume of 1 ml. The inoculum was loaded into a gastric feeding tube and inserted 5 cm into the rectum with the aid of lubricant. Animals were infused with 1 ml of the virus dilution followed by 3 ml of plain RPMI and 2 ml of air. All animals were exposed to virus dilution within 1 hr after the vial of stock virus was thawed.

### *S. mansoni* infections

Animals were anesthetized with ketamine and percutaneously exposed to 500 cercariae of a Puerto Rican strain of *S. mansoni*. An area on the abdomen was shaved, and cercariae were placed on the skin within a metal ring for 30 min to allow penetration. To monitor infection, fresh stool was obtained and processed by formalin-ethyl acetate sedimentation and concentration. Schistosome eggs were counted by microscopic examination. White blood cell counts (WBC) and percent eosinophils were calculated using standard methods. Hematology results, as well as CD4 and CD8 T-cell counts and ratios were determined by the Pathology Laboratory at Yerkes National Primate Research Center (Atlanta, GA). There was no evidence of fever, diarrhea, weight loss, or dysentery in these animals.

### Quantitation of viral RNA (vRNA) loads and simian cytokine mRNAs

Peripheral blood samples were obtained by venipuncture and collected into Vacutainer cell preparation tubes containing sodium citrate (Becton Dickinson, Rutherford, N.J.). Immediately after collection, plasma and PBMC were separated and quick frozen. Plasma samples from infected monkeys were stored at −80°C until vRNA loads were assessed by real-time RT-PCR [15]. Cells were washed once in RPMI 1640 (Gibco, Grand Island, NY), lysed with RLT lysis buffer (Qiagen, Valencia, CA) containing 1% β-mercaptoethanol (Sigma Chemical Company, St. Louis, MO), and stored at −80°C until they were processed to measure cytokine mRNA levels. A quantitative real-time RT-PCR assay based on TaqMan chemistry was utilized to measure mRNA levels for the cytokines interleukin IL-2, IL-4, IL-6, IL-10, IFN-γ, and TNF-α and the chemokine RANTES using previously described primers and protocols [16]. mRNA expression was normalized using primers unique for the housekeeping gene, phosphate dehydrogenase (PDH).

### Phenotyping and cell sorting

The following antibodies were used for both phenotyping and sorting of peripheral blood mononuclear cells (PBMC): CD3-Alexa700, CD4-PERCP-Cy5.5, CD8-APC-Cy7, CCR7-FITC, CD45RA-ECD, CD28-PE, CD95-APC; for cell sorting, we stained PBMC for CD4+ T-cell subsets only, using CD3, CD4, CD28 and CD95 mAbs. All reagents, except for CCR7 (R&D Systems, Minneapolis, MN), were obtained from BD Biosciences (San Jose, CA). For phenotyping and/or cell sorting, PBMC were isolated from blood collected and separated in Na citrate CPT tubes (Becton Dickinson). Cells were washed, counted, and resuspended in phosphate-buffered saline (PBS) containing 2% FCS with the appropriate mix of antibodies. For phenotyping, 2×10^6^ cells were stained, for cell sorting, a minimum of 10×10^6^ cells were evaluated. Following incubation with antibodies for 15 min under reduced light, cells were washed and resuspended in PBS containing 1% paraformaldehyde prior to analysis. Data were collected on a Becton Dickinson cell sorter FACSVantage with DiVa option (BD Biosciences) and analyzed with FlowJo analysis software (Tree Star, Inc., Ashland, OR). In both cases, we used an electronic gate on the forward scatter (FSC-H/FSC-A) to avoid doublets.

### Statistical analyses

Calculation of AID_50_ values and statistical comparison of infectious doses were performed using the method of Spouge [17]. Peak vRNA loads and single time point T-cell subsets were compared using the Wilcoxon rank-sum test. Statistical comparisons of vRNA loads and T-cell subsets over time between schistosome-infected and parasite-free groups were performed using repeated measures analysis. Modeling was performed with generalized estimating equations [18].

## Results

### *S. mansoni* infection significantly decreases the SHIV-C viral dose required to establish systemic infection

After preparation and storage of multiple vials of a large SHIV-C (strain SHIV-1157ipd3N4) stock, the minimal and AID_50_ necessary to establish systemic viral infection after mucosal challenge were determined in a group of 9 parasite-free animals (Table 1). Plasma vRNA loads were monitored prospectively by real-time RT-PCR (15). The minimal infectious dose for the parasite-free controls was 1 ml of virus stock diluted 1∶50; only 2 of the 3 animals exposed to this dilution became systemically infected. Based on the statistical method of Spouge [17], we determined that the AID_50_ of SHIV-C was 0.025 ml (95% confidence interval (CI), 7×10^−3^ to 8.7×10^−2^) for these control animals.

**Table 1:** SHIV-1157ipd3N4 titration.

Animal	Virus Dilution	Systemic infection	Peak viral RNA load (copies/ml×10^6^)	AID_50_	95% CI (*P*)
Parasite-free	Neat	+	1.3		
	1∶5·5*	+	2.1		
	1∶10	+	3.4		
	1∶50	+	8.2		
	1∶50	+	3.0		
	1∶50	−			
	1∶60	−			
	1∶100	−			
	1∶300	−			
	1∶1000*	−			
			Median: 3.0	1∶40	1∶11 to 1∶143
*S. mansoni*+	1∶50	+	20.6		
	1∶60	+	154.2		
	1∶100	+	70.3		
	1∶300	+	31.4		
	1∶300	+	34.6		
	1∶300	+	8.4		
	1∶500	−			
	1∶5000	−			
			Median: 33.0	1∶714	1∶200 to 1∶2500 (*P*<0.001)

*: This animal was first exposed to a 1∶1000 dilution of the virus. When it was not infected, the monkey was re-exposed to a 1∶5.5 dilution of virus, which was infective.

**Figure 1:**
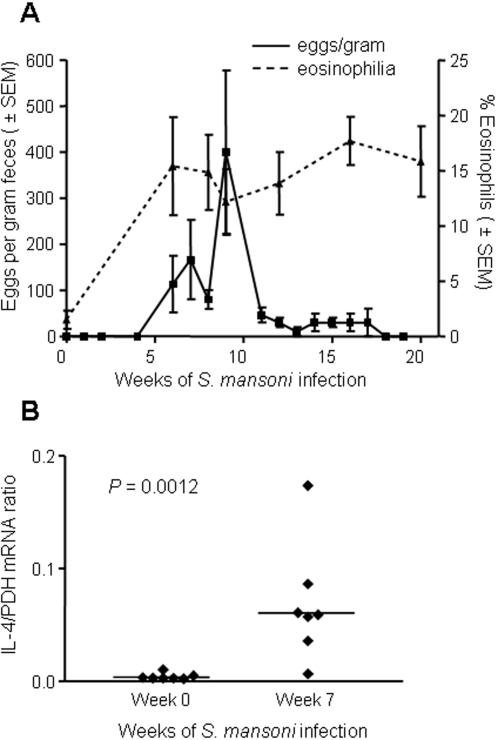
Parasitologic and immunologic changes in rhesus macaques infected with *Schistosoma mansoni*. (A) Eggs per gram feces in stool samples and percent eosinophils in blood from monkeys that were infected with *S. mansoni*; (B) IL-4 mRNA expression in PBMC of all *S. mansoni*-positive rhesus monkeys prior to exposure to SHIV-C. The ratios of IL-4 mRNA copies to mRNA copies of the housekeeping gene PDH are shown. Lines represent group medians. Statistical analysis of data in panel B was performed using the Wilcoxon rank-sum test.

To evaluate the impact of helminth infection on the susceptibility to SHIV-C, a second group of 8 macaques was infected with schistosomes by percutaneous exposure to 500 cercariae. Parasite infection was confirmed by measuring signs consistent with acute schistosomiasis: egg excretion in feces, eosinophilia (Figure 1A), and increased PBMC IL-4 mRNA levels (Figure 1B). In contrast, mRNA levels for IL-2, IL-6, IFN-γ, TNF-α, and RANTES did not differ between baseline and week 7 after infection (data not shown). Levels of IL-10 mRNA were increased as a result of schistosome infection at week 7 but were not statistically different. However, by 14 weeks of schistosome infection (7 weeks after exposure to SHIV) the increase in IL-10 mRNA was statistically significant (p = 0.047) compared to baseline. Monkeys were exposed to SHIV-C between 7 and 9 weeks after schistosome infection. The minimal infectious dose in animals with schistosomiasis was 1 ml of SHIV-C diluted 1∶300; all 3 animals exposed to this dose became systemically infected (Table 1). The AID_50_ for animals with schistosomiasis (0.0014 ml, 95% CI, 4×10^−4^ to 5×10^−3^) was 17-fold lower than that for parasite-free animals (*P*<0.001). The minimal infectious virus dose differed by a factor of 6 between the two groups of monkeys.

### *S. mansoni* infection significantly increases SHIV-C replication and host immune activation

**Figure 2:**
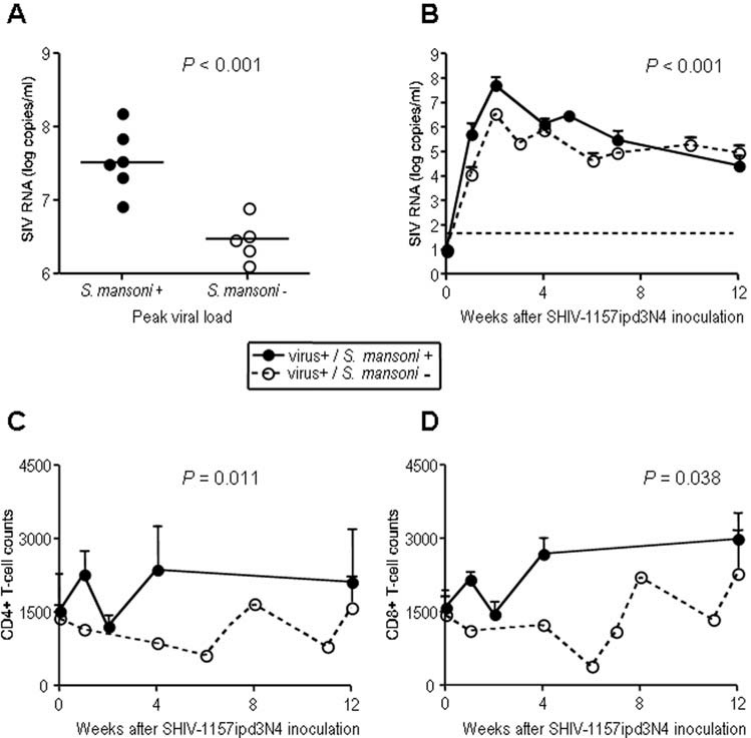
Virology and T cell subsets of schistosome infected and control animals following exposure to SHIV-C. (A) Peak vRNA loads (week 2 post-inoculation) and (B) longitudinal vRNA loads in coinfected and parasite-free monkeys. (C) CD4^+^ and (D) CD8^+^ T cells in coinfected and parasite-free monkeys. Lines in panel (A) represent group medians. For panels (B), (C) and (D), points represent group means and error bars represent standard deviations. Statistical analysis of data in panel A was performed using the Wilcoxon rank-sum test. Data in panels B–D were analyzed using repeated measures analysis and generalized estimating equations to compare the data over time for the two groups.

The mean peak vRNA load was >1 log higher in animals with acute schistosomiasis than in controls (*P*<0.001) even though the mean viral inoculum that led to systemic infection in animals with schistosomiasis (1∶185) was lower than that in parasite-free controls (1∶23) (Figure 2A). This is consistent with our previous findings that the magnitude of the peak vRNA load in infected animals depends on host factors and not on the viral concentration of the inoculum [19]. Elevated vRNA loads in parasite-infected animals were maintained through 10 weeks after virus exposure (*P*<0.001) (Figure 2B), similar to the elevated viral replication we observed in schistosome-infected animals that had been exposed intravenously to equal, high doses of a related SHIV [7].

Although vRNA loads were higher in animals with schistosomiasis, levels of both CD4^+^ (Figure 2C) and CD8^+^ (Figure 2D) T cells were also elevated in these animals compared to virus-only controls, consistent with the generalized immune activation caused by schistosomiasis. CD4/CD8 ratios were similar between the two groups and remained steady over time (data not shown).

### Increased viral replication in CD4+ central memory T cells of coinfected animals

**Figure 3:**
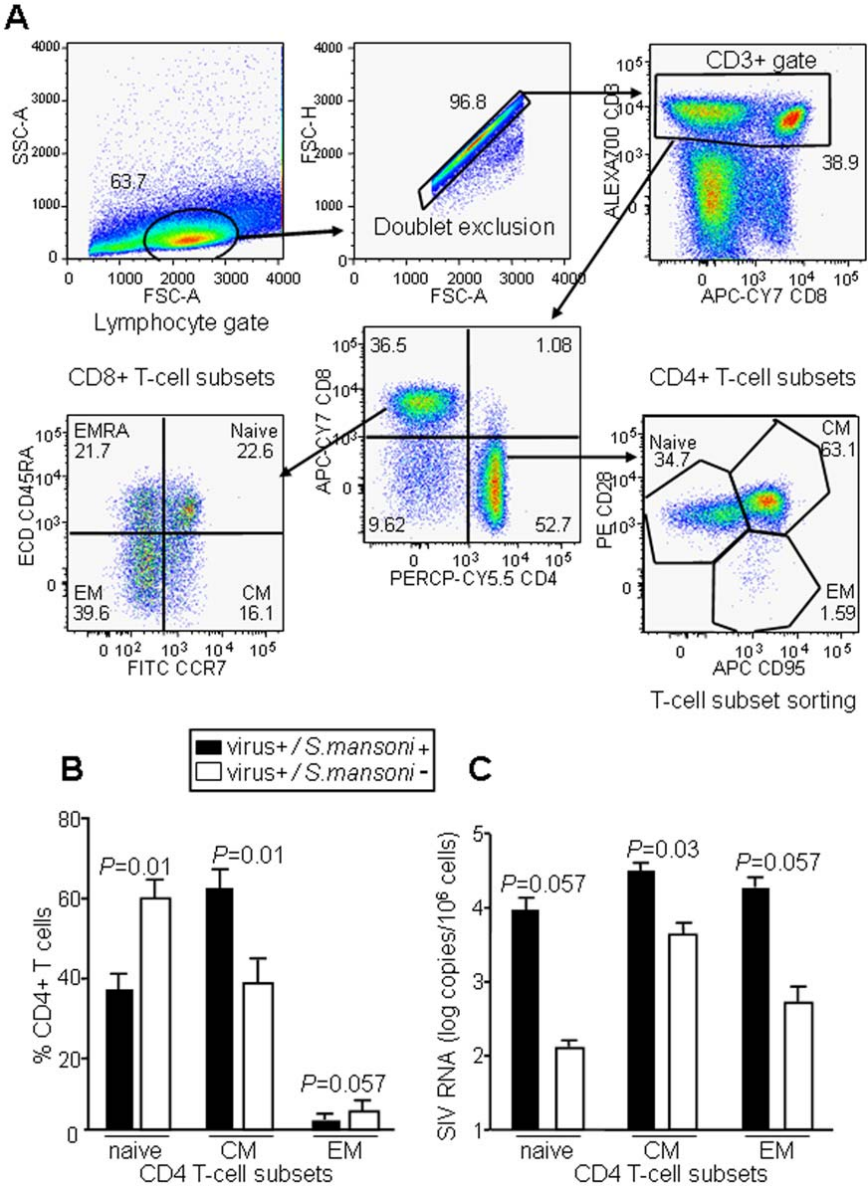
Differences in CD4^+^ T cell subsets and cell viral loads between animals with *S. mansoni*/SHIV-C coinfection and SHIV-C infection alone. (A) Phenotyping strategy used to separate naïve, central memory (CM), effector memory (EM) and effector memory CD45RA^+^ (EMRA) T cell subsets. Percentages demonstrate T cell subsets for one of the coinfected monkeys. (B) CD4^+^ naïve, CM and EM in coinfected and parasite-free monkeys. (C) SHIV-C RNA detected in CD4^+^ naïve, CM, and EM T cells after sorting. For panels (B) and (C), group means and standard deviations are represented.

PBMC from coinfected monkeys and from animals infected with SHIV-C alone were analyzed by multi-color flow cytometry after a month of viral inoculation (Figure 3A). The levels of CD4^+^ central memory (CM) T cells were significantly higher in coinfected animals, while parasite-free animals had more naïve CD4^+^ T cells (Figure 3B). We observed a similar pattern in CD8^+^ naïve and memory T cells but these differences were not statistically significant (data not shown).

Next, we sought to determine the prevalence of vRNA in different CD4^+^ T-cell subsets. We sorted CD4^+^ naïve and memory T-cell subsets for each group of animals (Figure 3A) and determined vRNA levels by RT-PCR (Figure 3C). Both naïve and memory CD4^+^ T cells from coinfected animals contained more vRNA per 10^6^ cells than the corresponding cells of animals infected with SHIV-C alone; the difference was statistically significant only in CD4^+^ CM subset (CD28^+^ CD95^+^) of T cells (*P* = 0.03) (Figure 3C). Consistent with the higher vRNA levels in CD4^+^ CM T cells of coinfected animals, 4 out of the 6 of these monkeys also had losses to abnormal levels (<10%) of the CD4^+^CD29^+^ subset of memory T-cells in peripheral blood, which is an early sign of immune dysfunction in lentivirus-infected-macaques [13],[20],[21]. In contrast, none of the 5 animals infected with virus alone fell below this threshold (data not shown). Together, these results suggest that CD4^+^ T cells from coinfected animals are more permissive to viral infection and replication, leading to more rapid destruction of memory T cells.

## Discussion

This study is the first direct evidence showing that helminth infection significantly increases host susceptibility to mucosal AIDS virus transmission in primates. Systemic infection of *S. mansoni*-infected rhesus macaques was established by low doses of virus that remained sub-infectious in parasite-free hosts, and the difference in viral dose needed to successfully establish systemic infection was surprisingly large. Furthermore, peak vRNA levels were also significantly elevated in schistosome-infected monkeys, consistent with previous in vitro, epidemiologic and primate model studies that suggest increased viral replication in schistosome-infected hosts or cells from persons with helminth infections [5]–[9]. However, none of the earlier studies was able to address whether schistosome infection increased the likelihood of *de novo* immunodeficiency virus infection in hosts harboring helminths. The increased susceptibility to mucosal immunodeficiency virus transmission we observed in schistosome-infected macaques, elevated viral replication in coinfected hosts, and accelerated loss of memory T cells all have profound public health implications for areas of the world where both parasitic worms and HIV-1 are endemic. Our data support the suggestion that better control of helminth infections may favorably impact efforts to reduce the spread of HIV/AIDS [3],[4].

Our observations are consistent with the hypothesis that helminth infections increase a hosts' susceptibility to infection with immunodeficiency viruses and is associated with a Th2-type immunologic phenotype and increased viral replication within these cells [22]. However, it is possible that any infection resulting in systemic immune activation or increased peripheral blood CD4^+^ T cell numbers (Figure 2C) would have yielded similar results [23]. Other data to support the hypothesis that Th2-type responses increase susceptibility to virus infection include the observation that PBMC from Kenyan schistosomiasis patients with HIV-1 coinfection produce decreased levels of IL-4 and IL-10 compared to patients with schistosomiasis alone. The magnitude of this effect correlates with the decrease in CD4^+^ T cells [24], suggesting that it is the CD4^+^ Th2 cells that are preferentially infected and killed by the virus. Alternatively, the shift to a predominant Th2-type response in schistosome-infected hosts may result in down-regulation of virus-controlling cytotoxic T lymphocytes [25]. We will evaluate these possibilities more directly in future studies.

We also considered whether the biology of parasite egg excretion, combined with the rectal route of viral exposure, may have increased host susceptibility to virus. Fecal egg excretion in hosts infected with *S. mansoni* or *S. japonicum* increases the number of activated T cells associated with egg granulomas that are in close juxtaposition to the intestinal lining [26]. Furthermore, the passage of eggs from mucosal tissue into the gut lumen may compromise the integrity of the epithelial lining, which in turn could lead to microbial translocation, release of immune-activating bacterial products into the bloodstream, and facilitate intrarectal SHIV-C transmission.

A limitation of our primate system is that we are only able to model the effect of the acute phase of schistosomiasis on susceptibility to viral transmission. This is because rhesus monkeys, while able to develop a fully patent infection, self cure their schistosomiasis at 20 to 25 weeks after exposure to cercariae [7],[8],[27]. Thus, we were not able to assess whether animals exposed to immunodeficiency virus during the chronic, more immunologically regulated phase of schistosome infection similarly display increased susceptibility to viral transmission. If the increased susceptibility to viral infection is related to the shift towards a Th2-type immune response, the increase in susceptibility to virus may be more modest during chronic infections when the phenotypic shift is less dramatic. Nevertheless, in human hosts with chronic schistosomiasis, both the immunologic stimulation and Th2-type responses (e.g., eosinophilia) persist. In addition, surface levels of the chemokine coreceptors CCR5 and CXCR4 are elevated on CD4^+^ T cells and monocytes of persons with chronic schistosomiasis and decrease following praziquantel treatment [28].

Our data strengthen the hypothesis that helminth infection may be a risk factor for increased susceptibility to de novo HIV-1 infection and support control of schistosomiasis and perhaps other helminths in persons living in areas endemic for these parasites. In the absence of an effective AIDS vaccine, there are several other strategies that may help decrease the risk of HIV-1 transmission, including control of sexually transmitted diseases [29] and male circumcision [30],[31]. Treatment of helminth infections is inexpensive, safe, and easily administered to large populations. In addition to the benefit of reducing host morbidity caused by the parasites, our data support control of helminths as a public health intervention for individuals at risk for acquiring HIV-1.

## References

[1] UNAIDS (2006). AIDS Epidemic update: December 2006.. http://data.unaids.org/pub/EpiReport/2006/2006_EpiUpdate_en.pdf.

[2] Morgan D, Whitworth J (2001). The natural history of HIV-1 infection in Africa.. Nat Med.

[3] Bentwich Z, Kalinkovich A, Weisman Z (1995). Immune activation is a dominant factor in the pathogenesis of African AIDS.. Immunol Today.

[4] Hotez PJ, Molyneux DH, Fenwick A, Ottesen E, Ehrlich Sachs S (2006). Incorporating a rapid-impact package for neglected tropical diseases with programs for HIV/AIDS, tuberculosis, and malaria.. PLoS Med.

[5] Shapira-Nahor O, Kalinkovich A, Weisman Z, Greenberg Z, Nahmias J (1998). Increased susceptibility to HIV-1 infection of peripheral blood mononuclear cells from chronically immune-activated individuals.. AIDS.

[6] Gopinath R, Ostrowski M, Justement SJ, Fauci AS, Nutman TB (2000). Filarial infections increase susceptibility to human immunodeficiency virus infection in peripheral blood mononuclear cells in vitro.. J Infect Dis.

[7] Chenine AL, Buckley KA, Li PL, Rasmussen RA, Ong H (2005). *Schistosoma mansoni* infection promotes SHIV clade C replication in rhesus macaques.. AIDS.

[8] Ayash-Rashkovsky M, Chenine AL, Steele LN, Lee SJ, Song R (2007). Coinfection with *Schistosoma mansoni* reactivates viremia in rhesus macaques with chronic simian-human immunodeficiency virus clade C infection.. Infect Immun.

[9] Kallestrup P, Zinyama R, Gomo E, Butterworth AE, Mudenge B (2005). Schistosomiasis and HIV-1 infection in rural Zimbabwe: effect of treatment of schistosomiasis on CD4 cell count and plasma HIV-1 RNA load.. J Infect Dis.

[10] Brown M, Mawa PA, Joseph S, Bukusuba J, Watera C (2005). Treatment of *Schistosoma mansoni* infection increases helminth-specific type 2 cytokine responses and HIV-1 loads in coinfected Ugandan adults.. J Infect Dis.

[11] Lawn SD, Karanja DMS, Mwinzi P, Andove J, Colley DG (2000). The effect of treatment of schistosomiasis on blood plasma HIV-1 RNA concentration in coinfected individuals.. AIDS.

[12] Elliott AM, Mawa PA, Joseph S, Namujju PB, Kizza M (2003). Associations between helminth infection and CD4+ T cell count, viral load and cytokine responses in HIV-1-infected Ugandan adults.. Trans R Soc Trop Med Hyg.

[13] Song RJ, Chenine AL, Rasmussen RA, Ruprecht CR, Mirshahidi S (2006). Molecularly cloned SHIV-1157ipd3N4: a highly replication-competent, mucosally transmissible R5 simian-human immunodeficiency virus encoding HIV clade C Env.. J Virol.

[14] Pope M, Haase AT (2003). Transmission, acute HIV-1 infection and the quest for strategies to prevent infection.. Nat Med.

[15] Hofmann-Lehmann R, Swenerton RK, Liska V, Leutenegger CM, Lutz H (2000). Sensitive and robust one-tube real-time reverse transcriptase-polymerase chain reaction to quantify SIV RNA load: comparison of one- versus two-enzyme systems.. AIDS Res Hum Retroviruses.

[16] Hofmann-Lehmann R, Williams AL, Swenerton RK, Li PL, Rasmussen RA (2002). Quantitation of simian cytokine and beta-chemokine mRNAs, using real-time reverse transcriptase-polymerase chain reaction: variations in expression during chronic primate lentivirus infection.. AIDS Res Hum Retroviruses.

[17] Spouge JL (1992). Statistical analysis of sparse infection data and its implications for retroviral treatment trials in primates.. Proc Natl Acad Sci U S A.

[18] Zeger SL, Liang KY (1986). Longitudinal data analysis for discrete and continuous outcomes.. Biometrics.

[19] Chenine AL, Ferrantelli F, Hofmann-Lehmann R, Vangel MG, McClure HM (2005). Older rhesus macaque infants are more susceptible to oral infection with simian-human immunodeficiency virus 89.6P than neonates.. J Virol.

[20] Sanders ME, Makgoba MW, Sharrow SO, Stephany D, Springer TA (1988). Human memory T lymphocytes express increased levels of three cell adhesion molecules (LFA-3, CD2, and LFA-1) and three other molecules (UCHL1, CDw29, and Pgp-1) and have enhanced IFN-γ production.. J Immunol.

[21] Baba TW, Jeong YS, Pennick D, Bronson R, Greene MF (1995). Pathogenicity of live, attenuated SIV after mucosal infection of neonatal macaques.. Science.

[22] Maggi E, Mazzetti M, Ravina A, Annunziato F, de Carli M (1994). Ability of HIV to promote a TH1 to TH0 shift and to replicate preferentially in TH2 and TH0 cells.. Science.

[23] Pentaleo G, Graziosi G, Fauci AS (1993). New concepts in the immunopathogenesis of human immunodeficiency virus infection.. N Engl J Med.

[24] Mwinzi PN, Karanja DM, Colley DG, Orago AS, Secor WE (2001). Cellular immune responses of schistosomiasis patients are altered by human immunodeficiency virus type 1 coinfection.. J Infect Dis.

[25] Actor JK, Shirai M, Kullberg MC, Buller RML, Sher A (1993). Helminth infection results in decreased virus-specific CD8^+^ cytotoxic T-cell and Th1 cytokine responses as well as delayed virus clearance.. Proc Natl Acad Sci USA.

[26] Damian RT (1987). The exploitation of host immune responses by parasites.. J Parasitol.

[27] McMullen DB, Ritchie LS, Oliver-Gonzalez J, Knight WB (1967). *Schistosoma mansoni* in *Macaca mulatta*. Long-term studies on the course of primary and challenge infections.. Am J Trop Med Hyg.

[28] Secor WE, Shah A, Mwinzi PM, Ndenga BA, Watta CO (2003). Increased density of human immunodeficiency virus type 1 coreceptors CCR5 and CXCR4 on the surfaces of CD4^+^ T cells and monocytes of patients with *Schistosoma mansoni* infection.. Infect Immun.

[29] Galvin SR, Cohen MS (2004). The role of sexually transmitted diseases in HIV transmission.. Nat Rev Microbiol.

[30] Bailey RC, Moses S, Parker CB, Agot K, Maclean I (2007). Male circumcision for HIV prevention in young men in Kisumu, Kenya: a randomised controlled trial.. Lancet.

[31] Gray RH, Kigozi G, Serwadda D, Makumbi F, Watya S (2007). Male circumcision for HIV prevention in men in Rakai, Uganda: a randomised trial.. Lancet.

